# Inflammatory Effects of Particulate Matter Exposure on the Nasal and Paranasal Sinus Mucosa in Rats

**DOI:** 10.3390/ijms26125885

**Published:** 2025-06-19

**Authors:** Hyun-Ho Kwak, Ji-Hwan Park, Hyang-Sook Kim, Hyun Min Lee, Sung-Dong Kim, Sue Jean Mun, Kyu-Sup Cho

**Affiliations:** 1 Department of Otorhinolaryngology, Pusan National University School of Medicine, Yangsan 50612, Republic of Korea; hanokwak@hanmail.net; 2 Department of Otorhinolaryngology and Research Institute for Convergence of Biomedical Science and Technology, Pusan National University Yangsan Hospital, Yangsan 50612, Republic of Korea; nobleivy@naver.com (J.-H.P.); enthmlee@gmail.com (H.M.L.); baskie23@naver.com (S.J.M.); 3 Research Institute for Convergence of Biomedical Science and Technology, Pusan National University Yangsan Hospital, Yangsan 50612, Republic of Korea; kesouk@hanmail.net; 4 Department of Otorhinolaryngology and Biomedical Research Institute, Pusan National University School of Medicine, Pusan National University Hospital, Busan 49241, Republic of Korea; applekims@daum.net

**Keywords:** particulate matter, nasal cavity, paranasal sinuses, mucous membrane, inflammation, rats

## Abstract

Particulate matter (PM) is a major environmental pollutant implicated in various respiratory diseases. However, its impact on the upper respiratory tract, particularly the nasal and paranasal sinus mucosa, remains poorly understood. This study aimed to investigate the acute inflammatory effects of PM exposure on the sinonasal mucosa and evaluate the natural recovery process in a controlled rat model. Ten-week-old male Sprague Dawley rats were exposed to incense-derived PM in a custom-designed exposure chamber for 2 h daily for seven consecutive days. Rats were sacrificed at 3, 7, and 14 days post-exposure. Histopathologic changes were assessed using hematoxylin and eosin and Alcian blue staining, and mucosal gene expression of inflammatory cytokines including interleukin (IL)-1β, IL-6, tumor necrosis factor (TNF)-α, interferon (IFN)-γ, and IL-5 and MUC5AC was quantified using real-time reverse transcription polymerase chain reaction. PM exposure induced significant histological alterations, including epithelial thickening, inflammatory cell infiltration, and goblet cell hyperplasia, which peaked at 7 days post-exposure. Expression levels of TNF-α and IFN-γ were significantly elevated at 7 days compared to controls. The sinonasal mucosa in the 14-day post-exposure groups exhibited a remarkable decrease in goblet cell numbers, and IL-1β and TNF-α expression. Short-term exposure to high concentrations of PM resulted in acute inflammatory changes in the sinonasal mucosa of rats, including epithelial thickening and goblet cell hyperplasia. These changes were partially resolved after exposure ended, indicating that PM-induced sinonasal inflammation may be at least partially reversible.

## 1. Introduction

Air pollution is a major global health concern, and particulate matter (PM) is one of the most harmful components [1]. PM consists of a complex mixture of solid and liquid particles suspended in the air and is typically categorized by size into PM_10_ (diameter ≤ 10 μm) and PM_2.5_ (diameter ≤ 2.5 μm) [2]. PM has been implicated in the development and exacerbation of cardiovascular diseases, Parkinson’s disease, Alzheimer’s disease, diabetes, and metabolic syndrome [3,4,5]. Furthermore, exposure to airborne PM has been associated with a range of respiratory diseases, including asthma, chronic obstructive pulmonary disease, and lung cancer [6,7,8]. PM triggers chronic inflammation in the airways, leading to airway hyperresponsiveness, increased mucus production, and reduced lung function [9].

The nasal cavity and paranasal sinuses serve as the first line of defense against inhaled pollutants by filtering, humidifying, and conditioning inspired air [10]. Chronic exposures to PM may disrupt these protective functions and contribute to the development of sinonasal inflammatory diseases such as chronic rhinosinusitis and allergic rhinitis [11,12]. Previous studies have suggested that PM can impair epithelial barrier integrity, induce inflammatory responses, and increase oxidative stress in the respiratory mucosa [13,14]. These effects are believed to occur through the disruption of tight junction proteins, including occludin and claudin-1, leading to increased mucosal permeability [15]. Furthermore, respiratory infections may also increase cytokine levels and induce mucosal inflammation. Notably, viral and bacterial infections differ in their immune profiles, with viral infections typically associated with increased IL-15 and natural killer cell activity, and bacterial infections with elevated IL-18 [16,17].

Several in vivo and in vitro studies have reported the impact of PM on the lower respiratory tract [18,19,20]. However, the effects of PM on the upper respiratory tract, particularly the nasal cavity and paranasal sinuses, remain less well understood, especially in controlled experimental models. Therefore, we developed a PM exposure model with controlled PM concentrations and investigated whether acute PM exposure induces inflammation in the nasal and paranasal sinus mucosa of rats by analyzing histological changes and cytokine expression. Furthermore, we evaluated the natural recovery process of mucosal inflammation over time following PM exposure.

## 2. Results

### 2.1. Histopathologic Analysis of Nasal and Paranasal Sinus Mucosa

**Figure 1:**
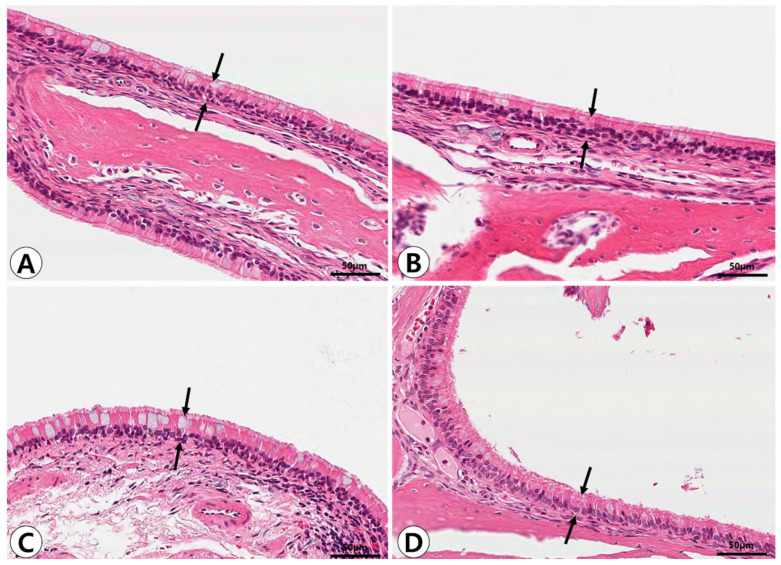
Histopathologic findings of sinonasal mucosa after particulate matter exposure. In the control group (**A**), the mucosal epithelium appears intact with no significant inflammatory cell infiltration. In the 3-day post-exposure group (**B**), mild epithelial thickening and inflammatory cell infiltration are observed. The 7-day post-exposure group (**C**) shows more pronounced epithelial thickening and inflammatory response. By day 14 post-exposure (**D**), partial recovery of the epithelial structure and a reduction in inflammation are noted. The thickness of the mucosal epithelium was measured at the sites indicated by black arrows in each panel (**A**–**D**) (hematoxylin and eosin, ×400).

**Figure 2:**
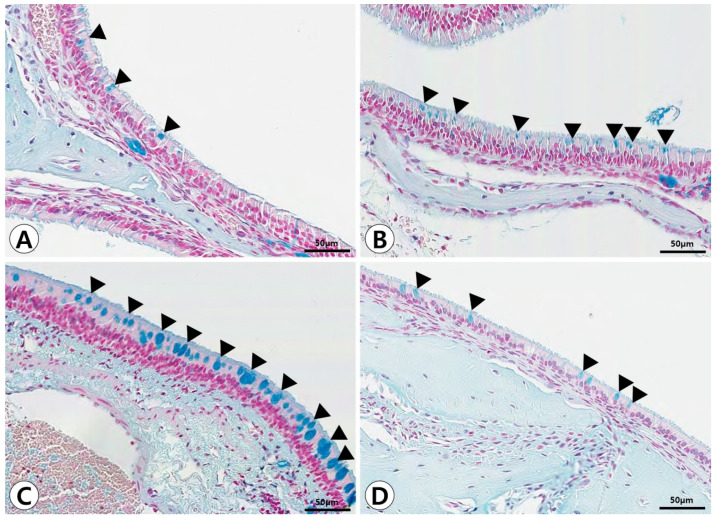
Goblet cell alterations in the sinonasal mucosa after particulate matter exposure. The control group (**A**) shows a normal distribution of goblet cells (arrowheads). In the 3-day post-exposure group (**B**), an increased number of Alcian blue-positive goblet cells is observed (arrowheads). The 7-day post-exposure group (**C**) exhibits marked goblet cell hyperplasia (arrowheads) and enhanced mucin production. By day 14 post-exposure (**D**), a partial reduction in goblet cell hyperplasia is noted (arrowheads) (Alcian blue stain, ×400).

Histopathological alterations in the nasal and paranasal sinus mucosa following PM exposure were evident in the groups exposed for 7 days. In the control group, the sinonasal mucosa exhibited a well-preserved pseudostratified mucociliary respiratory epithelium with goblet cells and no evidence of inflammatory cell infiltration. A significant increase in mucosal thickness and inflammatory cell infiltration was observed in the 7-day post-exposure group, indicating acute inflammatory changes. These alterations were reduced in the 14-day post-exposure group, suggesting partial recovery of the sinonasal mucosa (Figure 1). Goblet cell hyperplasia was determined by the increased number and size of goblet cells under Alcian blue staining, which was most prominent in the 7-day post-exposure group (Figure 2).

**Figure 3:**
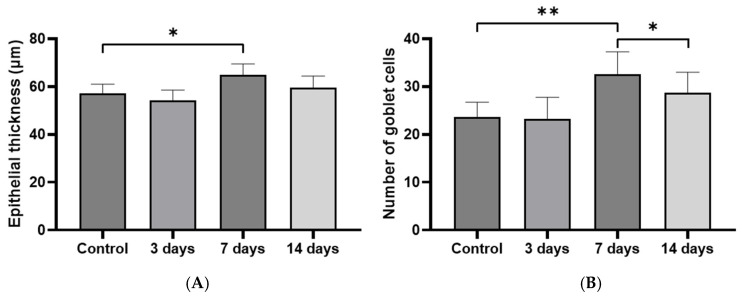
Quantitative analysis of epithelial thickness and goblet cell number in the sinonasal mucosa following particulate matter exposure: (**A**) A significant increase in mucosal thickness was observed in the 7-day post-exposure group compared to the control group, with partial recovery noted at day 14. (**B**) Goblet cell numbers significantly increased at 7 days post-exposure, followed by a significant decrease at day 14. Data are expressed as means ± standard deviation. * *p* < 0.05; ** *p* < 0.001.

In the quantitative analysis of epithelial thickness, the mean mucosal thickness was measured as follows: 57.19 ± 3.83 μm in the control group, 54.20 ± 4.34 μm in the 3-day post-exposure group, 64.85 ± 4.64 μm in the 7-day post-exposure group, and 59.59 ± 4.88 μm in the 14-day post-exposure group. A significant increase in mucosal thickness was observed in the 7-day post-exposure group compared to the control group (*p* = 0.002), with partial recovery noted at day 14. The number of goblet cells per high-power field (HPF) was significantly higher in the 7-day post-exposure group (32.67 ± 4.61) compared to the control group (23.67 ± 3.08) (*p* < 0.001). However, a significant reduction in goblet cell numbers was observed in the 14-day post-exposure group compared to the 7-day post-exposure group (*p* = 0.013), indicating resolution of the PM-induced hypersecretory response (Figure 3).

### 2.2. Expression of Inflammatory Cytokines and MUC5AC in the Sinonasal Mucosa

**Figure 4:**
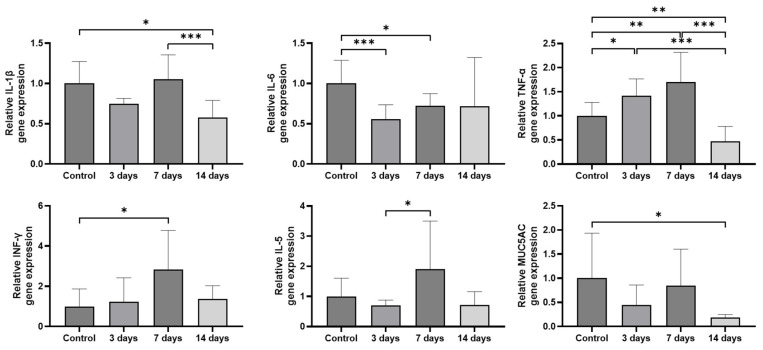
Expression of pro-inflammatory cytokines and MUC5AC in the sinonasal mucosa following particulate matter exposure. The expression of TNF-α and IFN-γ significantly increased (*p* = 0.008, *p* = 0.026, respectively) in the sinonasal mucosa of PM-exposed rats at 7 days post-exposure compared to the control group. MUC5AC expression showed an increasing trend in the 7-day post-exposure group compared to the 3-day post-exposure group. By 14 days post-exposure, the expression of IL-1β and TNF-α was remarkably decreased (*p* < 0.001). Data are expressed as means ± standard deviation. * *p* < 0.05; ** *p* < 0.01; *** *p* < 0.001.

The mRNA expression levels of pro-inflammatory cytokines and MUC5AC were analyzed using quantitative reverse transcription polymerase chain reaction (RT-PCR). Compared to the control group, the expression levels of TNF-α and IFN-γ were significantly increased by 1.70 ± 0.58 times (*p* = 0.008) and 2.84 ± 1.83 times (*p* = 0.026) in the sinonasal mucosa of PM-exposed rats at 7 days post-exposure, respectively. Although IL-1β and IL-5 expression showed an increasing trend, the difference did not reach statistical significance. Compared to the 3-day post-exposure group, the expression levels of IL-5 were significantly increased (*p* = 0.021) while IL-1β, IL-6, TNF-α, IFN-γ, and MUC5AC only showed an increasing trend in the sinonasal mucosa of PM-exposed rats at 7 days post-exposure. By 14 days post-exposure, expression levels of IL-1β and TNF-α were markedly reduced (*p* < 0.001) while IL-6, IFN-γ, IL-5, and MUC5AC only showed a decreasing trend compared to 7 days post-exposure, suggesting a resolution of PM-induced inflammation and secretory responses (Figure 4).

## 3. Discussion

PM contains a complex array of organic, inorganic, and organometallic compounds originating from both natural and anthropogenic combustion sources [21]. The chemical composition and cytotoxicity of PM may vary greatly depending on emission sources, atmospheric reactions, and regional environmental factors [22]. Furthermore, PM is classified by aerodynamic diameter into coarse (PM_10_), fine (PM_2.5_), and ultrafine (PM_0.1_) particles, each exhibiting distinct deposition patterns and biological effects in the respiratory tract [21]. Coarse PM, primarily generated through mechanical processes such as crushing and grinding, tends to deposit in the nasal cavity, oropharynx, and upper tracheobronchial regions. Fine PM_2.5_, originating mainly from combustion sources including vehicle emissions, coal burning, and industrial activity, can penetrate deep into the lungs, reaching the alveoli and entering both pulmonary and systemic circulations. Ultrafine particles, predominantly from vehicle exhaust, exhibit the highest potential for systemic translocation due to their ability to cross the alveolar–capillary barrier [23].

In this study, PM exposure was induced by burning incense sticks, a method that reflects real-world domestic and cultural PM sources. Although the particle size distribution was not measured directly, previous studies have consistently reported that incense combustion predominantly generates fine and ultrafine particles, with more than 70–90% of the total particle number concentration falling within the ultrafine range [24,25]. Incense burning produces a complex mixture of airborne pollutants, including PM, volatile organic compounds, polycyclic aromatic hydrocarbons, and various heavy metals such as lead, cadmium, and chromium [24]. These emissions can contribute to indoor air pollution levels that rival or exceed outdoor pollution in densely populated or poorly ventilated environments [26]. Previous studies have demonstrated that incense smoke induces oxidative stress and inflammatory responses in airway epithelial cells, making it a relevant, reproducible PM source for experimental models [25,27]. Although incense smoke may not fully replicate the chemical complexity of urban or traffic-related PM, its use in controlled experimental conditions provides a standardized and consistent means of evaluating the biologic effects of PM exposure on the sinonasal mucosa. Furthermore, a newly developed PM exposure model was used to deliver consistent and controlled levels of PM under reproducible environmental conditions [28]. This custom-designed chamber system, incorporating separate combustion and exposure chambers with an automated airflow control mechanism, allowed for the stable delivery of PM while maintaining constant temperature and humidity. In the present study, rats were exposed to average 24 h concentrations of 220.35 ± 20.94 μg/m^3^ for PM_10_ and 69.92 ± 5.35 μg/m^3^ for PM_2.5_, which substantially exceed the WHO 24 h air quality guidelines, thus allowing for the evaluation of sinonasal inflammatory responses under environmentally relevant high-exposure conditions.

Inflammatory responses induced by PM deposition in the respiratory tract have been demonstrated in both animal and human exposure studies. In vitro studies have demonstrated that PM exposure stimulates IL-6 production by alveolar macrophages, as well as TNF-α, IL-1β, granulocyte–macrophage colony-stimulating factor, and IL-8 by bronchial epithelial cells [18,19]. PM exposure resulted in epithelial thickening, mucous cell metaplasia, and infiltration of inflammatory cells, predominantly neutrophils, and macrophages in the nasal mucosa of rats [29]. Furthermore, PM_2.5_ exposure promoted oxidative stress and mitochondrial damage, suggesting that epithelial cell injury and dysfunction may play a key role in initiating or amplifying the inflammatory responses [30]. Controlled human exposure studies reported that short-term inhalation of PM leads to significant increases in IL-6 and TNF-α concentrations in bronchoalveolar lavage fluid and serum [31,32]. Although chronic exposure to PM, PM_2.5_ has been shown to induce sinonasal inflammation and epithelial barrier dysfunction in rodent models [33,34], the effects of acute PM exposure on the nasal and paranasal sinus mucosa, as well as the subsequent natural recovery process, remain to be elucidated. To the best of our knowledge, this is the first study to systematically investigate both the inflammatory response and spontaneous resolution of sinonasal mucosal changes following acute PM exposure in a controlled rat model.

The present study demonstrated that the short-term exposure to high concentrations of PM induces significant histological and molecular alterations in the nasal cavity and paranasal sinuses, including epithelial thickening, goblet cell hyperplasia, and upregulation of pro-inflammatory cytokines. The gene expression levels of TNF-α and IFN-γ were significantly increased following PM exposure. These cytokines are known to recruit neutrophils and macrophages, thereby amplifying the local inflammatory cascade. Additionally, increased expression of IL-5 implies an eosinophilic component of sinonasal inflammation. Furthermore, the elevated expression of MUC5AC, a major gel-forming mucin in the respiratory tract, is consistent with the goblet cell hyperplasia on histopathology and may represent an adaptive yet potentially pathological response to persistent mucosal irritation. However, MUC5AC can also be induced via IL-13 or epidermal growth factor receptor signaling [35,36]. Notably, these changes were more pronounced at 7 days post-exposure compared to 3 days, suggesting a progressive inflammatory and secretory response that peaks one week after PM exposure.

Importantly, our findings also revealed evidence of partial mucosal recovery following the cessation of PM exposure. The sinonasal mucosa in the 14-day post-exposure groups exhibited a gradual decline in inflammatory marker expression and histopathologic improvement, indicating that the sinonasal mucosa retains a certain degree of resilience. The decrease in goblet cell numbers observed in the 14-day post-exposure group suggests a spontaneous resolution of mucosal inflammation and hypersecretion following the cessation of particulate matter exposure. These findings support the concept that PM-induced sinonasal changes may be at least partially reversible, particularly after short-term exposure. However, repeated or prolonged exposure may exceed this regenerative capacity, potentially leading to irreversible tissue remodeling and chronic inflammation.

This study has several limitations. First, our study did not perform a direct chemical analysis of incense-derived PM. The PM used in this study was generated by incense combustion, which may not fully replicate the complex chemical composition of ambient urban air pollution, particularly traffic- and industry-related PM. Second, although this study demonstrated acute inflammatory responses and partial recovery, a time point immediately after the final PM exposure was not included, and the observation period was limited to 14 days post-exposure. The day 0 time point would help clarify when the inflammatory response begins and distinguish early changes from delayed effects. Long-term follow-up is necessary to determine the extent of full recovery or potential for chronic mucosal remodeling. Third, the analysis was restricted to histopathological and gene expression changes. Further studies incorporating protein-level confirmation through ELISA or immunohistochemical staining for TNF-α and MUC5AC and functional assessments such as mucociliary clearance would provide a more comprehensive understanding of PM-induced sinonasal dysfunction. Lastly, although the PM_10_ and PM_2.5_ exposure levels used in this study simulate severe pollution events, using a single concentration limits insight into dose-dependent effects. Future studies should include multiple exposure levels to clarify dose–response relationships and inflammatory thresholds.

## 4. Materials and Methods

### 4.1. Animals

**Figure 5:**
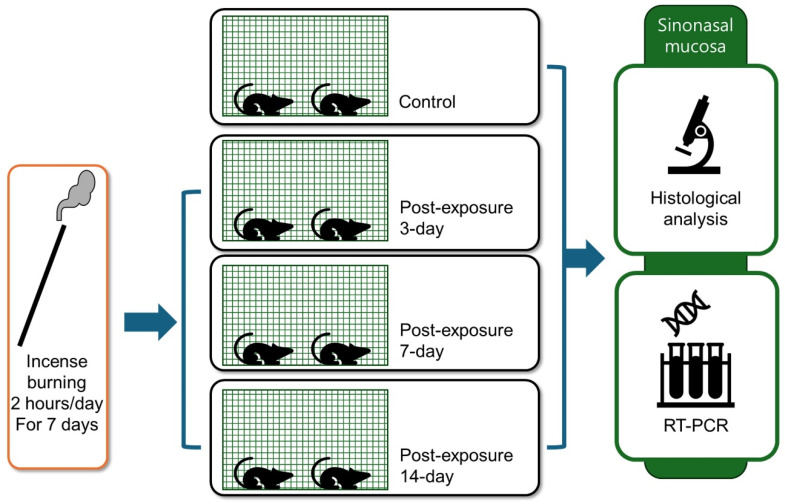
Schematic diagram of the experimental design. Rats were exposed to incense smoke for 2 h per day over 7 consecutive days. Four experimental groups were established: a control group with no exposure and three post-exposure groups (3 days, 7 days, and 14 days after the final exposure). Following the designated exposure or recovery periods, the animals were sacrificed, and nasal and paranasal sinus tissues were harvested for histological analysis and real-time reverse transcription polymerase chain reaction (RT-PCR) to evaluate inflammation-related changes.

Ten-week-old male Sprague Dawley rats weighing 250–300 g were purchased from Koatech Co. (Pyeongtaek, Republic of Korea) and bred in a specific pathogen-free (SPF) animal facility. To minimize potential confounding factors from infection, two rats were housed per cage to support animal welfare in a specific pathogen-free (SPF) environment with controlled conditions: temperature maintained at 20–24 °C, relative humidity between 40 and 60%, and alternating 12 h light–dark cycles. Prior to initiating experiments, animals underwent a 1-week quarantine period to screen for any underlying diseases. The rats were randomly allocated into four experimental groups (10 animals per group): control group, 3-day post-exposure group, 7-day post-exposure group, and 14-day post-exposure group. The chosen sample size was based on previous similar studies [28,37] and was deemed sufficient to detect meaningful differences in histopathologic and gene expression outcomes. During PM exposure, 3–4 rats were placed in the exposure chamber simultaneously. The experimental groups were exposed to PM for 7 days, then maintained for their designated waiting periods before being sacrificed for nasal tissue collection. The harvested nasal cavity and paranasal sinus tissues underwent histopathological examination using light microscopy and real-time RT-PCR analysis to evaluate changes in cytokine expression profiles (Figure 5). The animal study protocol was approved by the Institutional Animal Care and Use Committee of the Pusan National University Yangsan Hospital (Approval No. PNUYH IACUC-2023-025-A1C0).

### 4.2. PM Exposure Model

**Figure 6:**
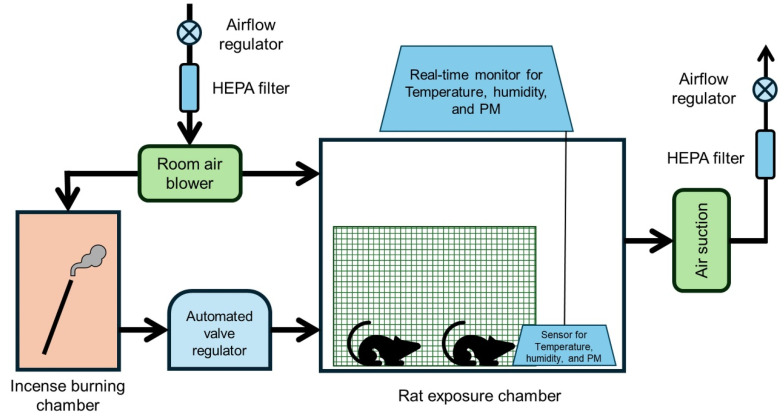
Schematic diagram of the particulate matter (PM) exposure model. A blower supplies room air to both the incense-burning chamber and the rat exposure chamber. Incense smoke generated in the burning chamber is directed into the rat exposure chamber through an automated valve regulator. Environmental conditions, including temperature, humidity, and PM concentration, are continuously monitored in real-time using embedded sensors within the exposure chamber. An air suction system is installed to ensure continuous ventilation of the chamber.

To ensure consistent PM exposure, a specialized chamber previously utilized by Lee et al. [26] was employed, in which PM was delivered while maintaining stable temperature and humidity conditions. Furthermore, an automatic control system was installed to regulate the opening between two connected chambers at predetermined intervals, thereby automating and standardizing the PM exposure process (Figure 6). The experimental setup consisted of two separate chambers: one for incense combustion as the PM source and the other for rat exposure. Ambient air was purified using a high-efficiency particulate air (HEPA) filter at a constant flow rate of 20 L/min to ventilate the rat exposure chamber, with the exhaust maintained at the same rate. The dimensions of the rat exposure chamber (0.45 × 0.6 × 0.4 m) were proportionally adjusted to match those of a standard bedroom (3.5 × 4.5 × 2.0 m) and human body weight, resulting in a total volume of 108 L [38]. The environmental conditions in the rat exposure chamber, including PM_10_, PM_2.5_, temperature, humidity, and carbon dioxide (CO_2_) levels, were continuously monitored using a measurement device.

PM was generated by burning 2 g of incense sticks (Dongshin-Hyang, PungNyun-Dang) for a duration of 2 h per day. Control group rats were placed in the exposure chamber for 2 h daily over 7 consecutive days without incense combustion. The experimental groups were exposed to PM for 2 h per day over a 7-day period. Temperature and humidity were maintained constant throughout the PM exposure period. Each experimental group was exposed to average concentrations of 2644.19 ± 251.29 μg/m^3^ of PM_10_ and 839.01 ± 63.75 μg/m^3^ of PM_2.5_ during the 2 h exposure sessions.

Following exposure, rats were returned to the SPF facility where PM levels were maintained at 0 μg/m^3^ through appropriate ventilation. Consequently, the rats experienced average 24 h exposure concentrations of 220.35 ± 20.94 μg/m^3^ for PM_10_ and 69.92 ± 5.35 μg/m^3^ for PM_2.5_, exceeding the WHO 24 h PM exposure guidelines by factors of 4.9 and 4.7, respectively. Sinonasal mucosa was harvested at 3, 7, and 14 days after the completion of the 7-day PM exposure period.

### 4.3. Histopathologic Analysis

Rats were sacrificed by decapitation following deep anesthesia induced by an overdose of isoflurane. After removing skin and soft tissues as well as the mandible of the head, the harvested skulls were fixed in 4% paraformaldehyde at 4 °C for 24 h. Subsequently, they were decalcified in a 10% ethylenediaminetetraacetic acid solution (pH 7.4) for about two months and then dehydrated with alcohol and xylene, embedded in paraffin. The paraffin blocks were sectioned coronally with a thickness of 4 μm. The selected sections extended from the level of incisive papilla to the second molar teeth, where ethmoid turbinates were most prominent. The true maxillary sinus and ethmoidal labyrinths were identified at the lesions posterior to the two maxillary turbinelles. Three consecutive coronal sections with similar sinus cavities were chosen for microscopic analyses. Hematoxylin and eosin (H & E) and Alcian blue staining were used to examine inflammatory cells and mucin-secreting cells, respectively. The epithelial thickness of the sinonasal mucosa and the number of goblet cells were determined by calculating the average of measurements taken at six distinct points within an HPF, specifically in the respiratory mucosa overlying the maxilloturbinates, where inflammatory changes were most reliably observed.

### 4.4. Analysis of Sinonasal Mucosa Using RT-PCR

Total RNA was extracted from the sinonasal mucosa of both control and experimental groups using QIAzol lysis reagent (Qiagen, Hilden, Germany). The extracted mRNA was reverse-transcribed into complementary DNA (cDNA) using Moloney murine leukemia virus reverse transcriptase (Promega, Madison, WI, USA). To evaluate the expression of IL-1β, IL-6, TNF-α, IFN-γ, IL-5, and MUC5AC, quantitative RT-PCR was performed in duplicate using the 2× real-time FastStart Essential DNA Green Master mix (Roche, Basel, Switzerland) according to the protocols of the manufacturer. GAPDH was used as a reference gene. The primers used for real-time PCR are listed in Table 1.

Expression levels were normalized against glyceraldehyde 3-phosphate dehydrogenase as an internal reference standard. The specific oligonucleotide primers employed in this study were adapted from previously published research (Table 1) [28,39].

### 4.5. Statistical Analysis

Data were expressed as means ± standard deviation. The Mann–Whitney U test was used to determine group differences, and all statistical analyses were conducted using the SPSS for Windows software package (version 28.0; SPSS Inc., Chicago, IL, USA). Graphs were generated using GraphPad Prism software (version 10.2.3; GraphPad Software, Inc., San Diego, CA, USA). A value of *p* < 0.05 was considered to indicate statistical significance.

## 5. Conclusions

Short-term exposure to high concentrations of PM induced acute inflammation, epithelial thickening, and goblet cell hyperplasia in the sinonasal mucosa of rats. These changes showed partial recovery over time after cessation of exposure, suggesting a reversible inflammatory response to PM.

## Figures and Tables

**Table 1:** Polymerase chain reaction primer sequences for the rats used in this study.

	Sequence (5′–3′)		Product Size (bp)
IL-1ß	Forward	GCAATGGTCGGGACATAGTTGA	158
	Reverse	AGACCTGACTTGGCAGAGGA	
IL-6	Forward	ACCCCAACTTCCAATGCTCT	135
	Reverse	GGTTTGCCGAGTAGACCTCA	
TNF-a	Forward	ACCACGCTCTTCTGTCTACTG	170
	Reverse	TGCTTGGTGGTTTGCTACGAC	
INF-γ	Forward	GGCAAAAGGACGGTAACAC	200
	Reverse	GTTGTTCACCTCGAACTTGG	
IL-5	Forward	CAATGAGACGATGAGGCTTC	175
	Reverse	CACTTCTCTTTTTGTCCGTCAA	
MUC5AC	Forward	ACTATGAGGTGCGACTGCTT	158
	Reverse	CTTGTGGGATGTCACAGGAGT	
GAPDH	Forward	GATGGTGAAGGTCGGTGTGA	163
	Reverse	GAACTTGCCGTGGGTAGAG	

IL, interleukin; TNF, tumor necrosis factor; INF, interferon; GAPDH, glyceraldehyde 3-phosphate dehydrogenase.

## Data Availability

The data used to support the findings of this study are available from the corresponding author upon request.
